# The application of spatial measures to analyse health service accessibility in Australia: a systematic review and recommendations for future practice

**DOI:** 10.1186/s12913-023-09342-6

**Published:** 2023-04-01

**Authors:** Sarah M. Wood, Laura Alston, Hannah Beks, Kevin Mc Namara, Neil T. Coffee, Robyn A. Clark, Anna Wong Shee, Vincent L. Versace

**Affiliations:** 1 School of Medicine, Faculty of Health, Deakin Rural Health, Deakin University, Warrnambool Campus, PO Box 423, Warrnambool, VIC 3280 Australia; 2 Research Unit, Colac Area Health, Colac, Vic Australia; 3 Grampians Health, Ballarat, Vic Australia; 4 University of Canberra, Canberra, ACT Australia; 5 Caring Futures Institute, Flinders University, Adelaide, SA Australia

**Keywords:** Health service, Access, Spatial access, Geography, Spatial analysis, GIS, Systematic review, Delivery of healthcare, Health equity

## Abstract

**Background:**

Australia's inequitable distribution of health services is well documented. Spatial access relates to the geographic limitations affecting the availability and accessibility of healthcare practitioners and services. Issues associated with spatial access are often influenced by Australia's vast landmass, challenging environments, uneven population concentration, and sparsely distributed populations in rural and remote areas. Measuring access contributes to a broader understanding of the performance of health systems, particularly in rural/remote areas. This systematic review synthesises the evidence identifying what spatial measures and geographic classifications are used and how they are applied in the Australian peer-reviewed literature.

**Methods:**

A systematic search of peer-reviewed literature published between 2002 and 2022 was undertaken using the Preferred Reporting Items for Systematic Reviews and Meta-Analyses (PRISMA) methodology. Search terms were derived from three major topics, including: [1] Australian population; [2] spatial analysis of health service accessibility; and [3] objective physical access measures.

**Results:**

Database searches retrieved 1,381 unique records. Records were screened for eligibility, resulting in 82 articles for inclusion. Most articles analysed access to primary health services (*n* = 50; 61%), followed by specialist care (*n* = 17; 21%), hospital services (*n* = 12; 15%), and health promotion and prevention (*n* = 3; 4%). The geographic scope of the 82 articles included national (*n* = 33; 40%), state (*n* = 27; 33%), metropolitan (*n* = 18; 22%), and specified regional / rural /remote area (*n* = 4; 5%). Most articles used distance-based physical access measures, including travel time (*n* = 30; 37%) and travel distance along a road network (*n* = 21; 26%), and Euclidean distance (*n* = 24; 29%).

**Conclusion:**

This review is the first comprehensive systematic review to synthesise the evidence on how spatial measures have been applied to measure health service accessibility in the Australian context over the past two decades. Objective and transparent access measures that are fit for purpose are imperative to address persistent health inequities and inform equitable resource distribution and evidence-based policymaking.

**Supplementary Information:**

The online version contains supplementary material available at 10.1186/s12913-023-09342-6.

## Background

Health inequities are unjust differences in health risks and outcomes due to economic, social, political, and cultural disadvantages [1]. They are a significant challenge globally and require substantial policy investment in areas such as employment, education, housing, transport, and access to health services across different areas [2]. Consistent spatial measures and geographic classifications are required to meaningfully identify and compare geographic areas, inform decision-making, and develop health policies to address inequities.

Access to healthcare is a critical measure of healthcare systems’ performance and directly impacts population health and disease burden. For example, evidence shows that improving access to primary care leads to better health outcomes and decreases potentially avoidable hospitalisations [2–7]. Access is a complex yet important concept in health service and policy research, defined in both spatial and aspatial terms [8–10]. Aspatial access concerns the non-geographic factors affecting access, such as affordability, timeliness, accommodation, acceptability, and awareness [10, 11]. Spatial access relates to geographic factors affecting the availability and accessibility of healthcare providers and services [11, 12]. Identifying areas with limited spatial accessibility enables planners and policymakers to understand the distribution of health service locations to address spatial inequities [3, 13, 14].

There are multiple methods for measuring access; for example, spatial access measures can be area-based or distance-based [15]. Area-based measures are crude measures that refer to towns, cities, or states. The provider-to-population ratio (PPR) is commonly used to calculate the supply ratio within an area. PPRs are easily interpreted indicators readily understood by policymakers; however, they are often subject to the modifiable areal unit problem (MAUP) due to the fixed geographic or administrative boundaries, such as local government areas and postcodes [15–17]. Distance access measures focus on distance or travel time. Distance results can vary depending on the methods used; for example, simple distance metrics, such as straight-line Euclidean distance or the more sophisticated, network distances. Euclidean distance is simply the straight-line distance between two points. In contrast, network distance is the shortest route between two points in a spatial network and can account for variables such as travel time along a road network. In health service research, distance calculations can estimate the travel time to a healthcare facility from a given locality or calculate the travel cost [13].

Currently, there is no agreed definition or national policy on what constitutes reasonable access to health services in Australia regarding the maximum time or distance a person needs to travel for healthcare [18]. For prehospital management of major trauma in Australia, guidelines vary across each state; for example, New South Wales (NSW) guidelines list travel time within 60-min for metropolitan and 90-min for regional, while Victoria uses a 45-min transport time [19, 20]. Research has highlighted the types of spatial measures of accessibility used in policymaking and the importance of these measures. Dewulf et al. [21] observed substantial variations in measured spatial distribution and accessibility depending on the methods applied, highlighting the importance of appropriate access measures at a policy level [21].

Historically, there has been heterogeneity in how Australian research reports geographic classifications and defines populations, such as rural and remote populations [22–24]. A systematic review by Alston et al. [22] on the burden of cardiovascular disease (CVD) in Australia found that some rural and urban areas could not be compared due to the heterogeneity of methods and geographic classifications, consequently, the authors could not provide a clear conclusion on the level of disparity in CVD outcomes by remoteness. Similarly, Beks et al. [23] also reported variations in the use of geographical classification approaches to defining rurality, including systems that are no longer meaningful to policymakers and adaptations of existing systems that make study comparisons difficult.

To address health inequity in Australia, it is crucial to understand how health service access is measured, and currently, it is unclear what measures are being used. A lack of analysis of existing measures inhibits the ability to assess the appropriateness of access measures in line with national policy. To our knowledge, no comprehensive systematic review has been undertaken to synthesise the peer-reviewed literature measuring health service accessibility in Australia. Synthesis is needed to understand and identify current gaps and make recommendations for future research that will inform action on the maldistribution of health services to address health inequities, especially for rural and remote areas. This systematic review aims to address this knowledge gap by answering the question: *What spatial measures are being applied to examine health service accessibility in Australia?*

## Methods

This systematic review followed the Preferred Reporting Items for Systematic Reviews and Meta-Analyses (PRISMA) statement [25]. The PRISMA checklist is available is the supplementary materials (see Supplementary file 1). A protocol for this review was developed in advance and registered in PROSPERO (CD: CRD42022302108).

### Eligibility criteria

The PICOS mnemonic was used to develop the search criteria and study eligibility (Table 1). *Population* includes articles that report data from a subset of the Australian population; *Intervention* was the examination of spatial access to a health service; *Outcomes* include the objective physical access measures and spatial analyses used in articles. Articles were included in the review if they were published in English in a peer-reviewed journal between 1 January 2002 and 14 March 2022. The past twenty years were chosen to align with the development of spatial research in Australia [26]. Grey literature was excluded from this review.

**Table 1:** PICOS

CRITERIA	INCLUSION	EXCLUSION
**P (Population)**	• Australia	• Outside Australia
**I (Intervention)**	• Spatial access of health services, including primary healthcare, specialist care, hospital services, and cancer screening services	• No focus on health service access
**C (Comparison)**	• none	
**O (Outcomes)**	• *Primary Outcome Measure:* objective physical access measures • *Secondary Outcome Measure:* spatial analysis applied	• No objective physical access measures
**S (Study design)**	• Epidemiological observational articles that include geographic or ecological-level data with spatial analysis	
**Time period**	• 2002–2022	

### Information sources

Database searches were completed on 14 March 2022, including MEDLINE, Embase (Elsevier), Scopus, CINAHL (EBSCOhost), Web of Science, Global Health (EBSCOhost), and Environmental Complete (EBSCOhost). Additional to database searching, reference lists of included articles were reviewed for relevant articles.

### Search strategy

Search terms were developed from an initial limited search of MEDLINE and CINAHL and reviewing relevant literature. The keywords contained in the titles and abstracts of relevant articles and the MeSH terms used to describe the articles were used to develop the full search strategy. A combination of search terms was used, such as “general practi*” OR doctor OR hospitals OR “emergency department” AND “geographic* information system*” OR spatial. A supplementary file outlines the complete search strategies (see Supplementary file 2). Two librarians with expertise in developing search strategies for databases reviewed the searches. The search strategy, including all identified keywords and index terms, was adapted for each included database.

### Screening and selection

**Fig. 1:**
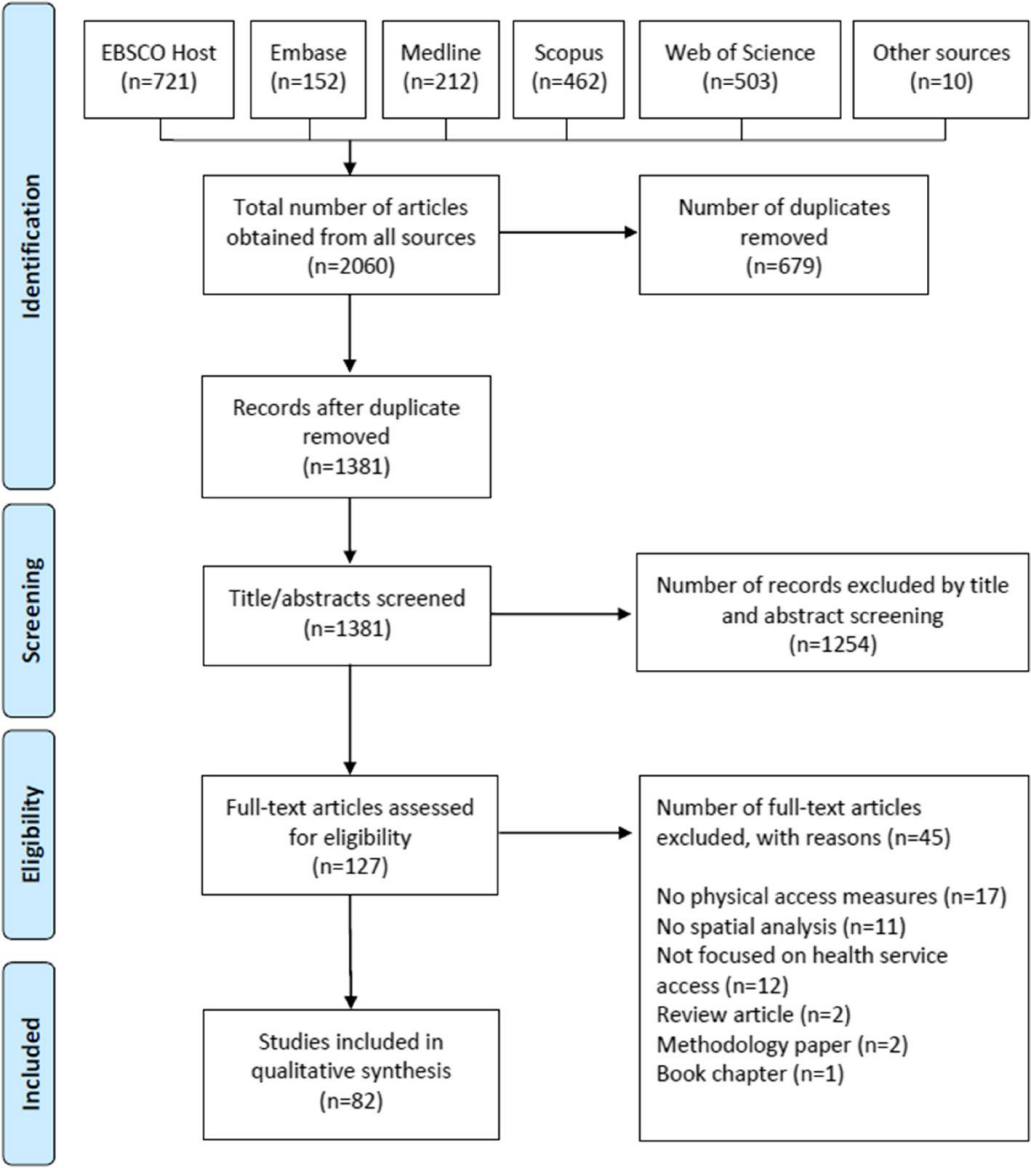
PRISMA flow diagram of screening process

Following the search, all identified citations were collated and uploaded into Endnote (Version 20.2.1, Clarivate, Philadelphia, PA). Citations were imported into Covidence (Veritas Health Innovation, Melbourne, Australia), and duplicates were removed. Titles and abstracts were screened in Covidence by three independent reviewers (SW, HB, and LA) for assessment against the eligibility criteria for the review. Potentially relevant articles were retrieved as full texts and assessed in detail against the inclusion criteria by SW, LA, and HB. Reasons for exclusion at the full-text stage were recorded. Any disagreements between the reviewers during the selection process were resolved through discussion between SW, LA, and HB. The results of the full search and the article inclusion process are presented in a Preferred Reporting Items for Systematic Reviews and Meta-analyses (PRISMA) flow diagram (Fig. 1).

### Data extraction

Data were extracted from articles by SW using a pre-determined data extraction tool developed by the reviewers. To ensure quality, ten per cent of the extracted data was crossed checked by another reviewer (LA). The data extracted used a pre-determined template and included specific details about the study population, context, type of health service, study methods, geographic classification and spatial analyses used, and key findings relevant to the review question.

### Quality assessment

Quality appraisal tools are less established in ecological research than in reviews of randomised controlled trials. As there is no existing accepted quality assessment tool for health geography research, this systematic review used the Joanna Briggs Institute analytical cross-sectional studies critical appraisal checklist [27]. The checklist consists of eight items, with a choice of ‘yes’, ‘no’, ‘unclear’, or ‘not applicable’. Quality appraisal was completed by SW and to ensure quality, ten per cent of the articles were crossed checked by another reviewer (LA). Any disagreements between the reviewers were resolved through discussion between SW, LA, and HB.

### Data synthesis

A narrative synthesis of the results was conducted, including the geographic scope and health service context for the articles. Health services were classified and presented according to the Australian Institute of Health and Welfare (AIHW) definitions (Table 2) [28]. Due to the overlap of some health services, such as specialist care and hospitals, this review defined specialist care as any service for those with specific or complex conditions or issues, such as cancer treatment or cardiac rehabilitation services. The primary and secondary outcome measures were synthesised, including the objective physical access measures and spatial models applied to understand service access in Australia. Quantitative synthesis was not undertaken due to the methodological heterogeneity of the articles included in this review.

**Table 2:** Types of health services in Australia

Health services	Definition	Example of services
Health promotion and prevention	Improving health and preventing ill health	• Immunisation and vaccination • Cancer screening • Disease prevention programs
Primary healthcare	First contact with the health system	• General practitioner • Allied health • Pharmacy • Community health
Specialist care	Provides services for those with specific or complex conditions or issues	• Mental health services • Cancer treatment • Alcohol and other drug treatment services • Palliative care • Diagnostic services • Referred medical specialist services
Hospitals	Services provided to admitted and non-admitted patients	• Inpatient • Outpatient clinics • Emergency department care

Source: Adapted from AIHW [28]

## Results

Searches retrieved 1,381 unique citations, which were screened for inclusion based on their title and abstract (Fig. 1). Of these, the full texts of 127 articles were reviewed, with 82 articles meeting the inclusion criteria. Reasons for exclusion included: no objective physical access measures (*n* = 17); no spatial analysis (*n* = 11); not focused on health services access (*n* = 12); and methodology paper (*n* = 2), case studies or review articles (*n* = 3).

### Characteristics of selected articles

The characteristics of the 82 articles included are presented in Table 3. Most articles analysed access to primary health services (*n* = 50; 61%), followed by specialist care (*n* = 17; 21%), hospital services (*n* = 12; 15%), and health promotion and prevention (*n* = 3; 4%). Articles utilised various data sources to analyse target populations, including census data (*n* = 61), patient databases or records from individual health services (*n* = 14), national databases (*n* = 5), state databases or registries (*n* = 14), survey data (*n* = 6), and research databases (*n* = 3). Articles that analysed the location of health service locations, used sources such as publicly available databases (*n* = 22), state departments (*n* = 14), National Health Services Directory (*n* = 9), Medical Directory Australia (*n* = 5), surveys (*n* = 5), and Points of Interest portfolios (*n* = 5). Articles that included provider information used sources such as national databases, including membership registries (*n *= 3) and the Australian Health Practitioner Regulation Authority (*n* = 2).

**Table 3:** Summary table of included articles (*n *= 82)

First author, reference	Year	Health service area	Health discipline	Location: spatial unit	MMM	Spatial access measures	Quality Appraisal ^a^							
							**1)**	**2)**	**3)**	**4)**	**5)**	**6)**	**7)**	**8)**
Almado [29]	2015	Primary health	Dental services	City: CD	MM 1	Distance	Y	U	Y	Y	Y	N	Y	Y
Alsharif [30]	2016	Primary health	Dental services	City: CD	MM 1	Distance	Y	Y	Y	Y	Y	N	Y	Y
Bray [31]	2017	Hospitals	Emergency care	State: PHN	MM 1–7	Travel time	Y	U	Y	Y	N	N	Y	Y
Carman [32]	2010	Primary health	Other primary care services	National: PC	MM 1–7	Distance	Y	Y	Y	Y	Y	N	Y	U
Chong[33]	2015	Primary health	GP services	City: CD	MM 1	Road distance	Y	Y	Y	Y	Y	Y	Y	Y
Clark [34]	2009	Specialist care	Cardiac services	National: CD	MM 1–7	*n* of services	Y	Y	Y	Y	N	N	Y	U
Clark [35]	2007	Specialist care	Cardiac services	National: CD	MM 1–7	Road distance	Y	Y	Y	Y	N	N	Y	Y
Clark [36]	2012	Hospitals	Acute cardiac care	National: CD	MM 1–7	Travel time Road distance	Y	Y	Y	Y	NA	NA	Y	Y
Clark [37]	2014	Specialist care	Cardiac services	National: CD	MM 1–7	Travel time *n* of services	Y	Y	Y	Y	NA	NA	Y	Y
Coffee [38]	2012	Hospitals	Acute cardiac care	National: CD	MM 1–7	Road distanceTravel time	Y	Y	Y	Y	NA	NA	Y	Y
Cramb [39]	2012	Specialist care	Cancer treatment	State: SLA	MM 1–7	Travel time	Y	Y	Y	Y	Y	Y	Y	Y
Currow [40]	2012	Specialist care	Palliative care	National: PC	MM 1–7	Distance	Y	Y	Y	Y	Y	Y	Y	Y
Dudko [41]	2017	Primary health	Dental services	National: SA2	MM 1–7	Distance	Y	U	Y	Y	N	N	Y	Y
Edirippulige [42]	2016	Hospitals	Outpatient clinic	State: PC	MM 1–7	DistanceTravel time	Y	Y	Y	Y	N	N	Y	Y
Evans [43]	2017	Primary health	GP services	Regional Centre: SA1	MM 2	Distance	Y	Y	Y	Y	Y	N	Y	Y
Flabouris [44]	2012	Hospitals	Intensive care services	National: PC	MM 1–7	Distance	Y	Y	Y	Y	Y	N	Y	Y
Flabouris [45]	2012	Hospitals	Intensive care services	National: PC	MM 1–7	Distance	Y	Y	Y	Y	Y	Y	Y	Y
Gabriel [46]	2015	Specialist care	Cancer treatment	State: MB	MM 1–7	Distance	Y	Y	Y	Y	Y	Y	Y	Y
Gao [47]	2019	Primary health	Allied Health	City: SA2	MM 1	No of services	Y	Y	Y	Y	N	N	Y	Y
Gardiner [48]	2020	Primary health	Dental services	National: SA3	MM 2–7	Travel time	Y	Y	Y	Y	Y	N	Y	Y
Gardiner [49]	2020	Specialist care	Renal disease management services	National: SA3	MM 2–7	Distance Travel time	Y	Y	Y	Y	Y	N	Y	Y
Giummarra [50]	2021	Hospitals	Outpatient clinic	State: PC	MM 1–2	Travel time	Y	Y	Y	Y	Y	Y	Y	Y
Gomez [51]	2019	Hospitals	Emergency care	State: SA1	MM 1–7	Travel time	Y	Y	Y	Y	N	N	Y	Y
Graham [52]	2019	Primary health	Dental services	National: SA2	MM 1–7	*n* of practices	Y	U	Y	Y	Y	U	Y	Y
Homer [53]	2011	Hospitals	Maternity services	National: ASGS-RA	MM 1–7	*n* of services	Y	U	Y	Y	Y	N	Y	Y
Hsieh [54]	2015	Specialist care	Cancer treatment	State: SLA	MM 1–7	Travel time	Y	Y	Y	Y	Y	Y	Y	Y
Hsieh [55]	2016	Specialist care	Cancer treatment	State: SLA	MM 1–7	Distance	Y	Y	Y	Y	Y	Y	Y	Y
Hyndman [56]	2003	Primary health	GP services	City: CD	MM 1	Distance	Y	Y	Y	Y	Y	Y	Y	Y
Jean [57]	2019	Primary health	Dental services	National: SA2	MM 1–7	PPR	Y	Y	Y	Y	Y	N	Y	Y
Jean [58]	2020	Primary health	Dental services	National: SA2	MM 1–7	PPR	Y	Y	Y	Y	Y	N	Y	Y
Jean [59]	2020	Primary health	Dental services	National: SA1	MM 1–7	*n* of providersTravel time	Y	Y	Y	Y	Y	N	Y	Y
Jean [60]	2020	Primary health	Dental services	National: SA2	MM 1–7	PPR	Y	Y	Y	Y	N	N	Y	Y
Kamil [61]	2022	Primary health	Dental services	National: SA1	MM 1–7	*n* of practices	Y	Y	Y	Y	Y	N	Y	Y
Khan [62]	2021	Health promotion & protection	Cancer screening	City: SSC	MM 1	Distance	Y	Y	Y	Y	Y	Y	Y	Y
Khan [63]	2021	Health promotion & protection	Cancer screening	City: SSC	MM 1	Distance	Y	Y	Y	Y	Y	Y	Y	Y
Kloot [64]	2016	Hospitals	Emergency care	Region: LGA	MM 2–6	Distance	Y	Y	Y	Y	N	N	Y	Y
Kruger [65]	2011	Primary health	Dental services	State: CD	MM 1–7	*n* of practices Distance	Y	U	Y	Y	Y	N	Y	Y
Lakhani [66]	2021	Health promotion & protection	Infectious disease screening	State: MB	MM 1–6	Travel time	Y	Y	Y	Y	Y	N	Y	Y
Lakhani [67]	2020	Specialist care	Palliative care	City: SA1	MM 1	Travel time	Y	Y	Y	Y	N	N	Y	Y
Lakhani [68]	2021	Primary health	Aboriginal and Torres Strait Islander health services	State: SA2	MM 1–7	Travel time	Y	Y	Y	Y	N	N	Y	Y
Lakhani [69]	2019	Primary health	Allied Health	State: SA2	MM 1–7	Travel time	Y	Y	Y	Y	Y	Y	Y	Y
Le [70]	2019	Primary health	Pharmacy	State: PC	MM 1–7	Distance Travel time	Y	Y	Y	Y	N	N	Y	Y
Liu [71]	2021	Primary health	Dental services	National: SA1	MM 1	Distance *n* of practices	Y	U	Y	Y	N	N	Y	Y
Madill [72]	2018	Primary health	Other primary care services	City: LGA	MM 1	Travel time	Y	Y	Y	Y	Y	N	Y	Y
Mazumdar [73]	2020	Primary health	GP services	City: POAs	MM 1	PPR	Y	Y	Y	Y	Y	Y	Y	Y
McCormack [74]	2015	Primary health	Allied Health	National: PC, LGA	MM 1–7	PPR	Y	Y	Y	Y	N	N	Y	Y
McGrail [75]	2009	Primary health	GP services	State: CD	MM 2–6	*n* of services PPR Travel time	Y	Y	Y	Y	NA	NA	Y	Y
McGrail [76]	2009	Primary health	GP services	State: CD	MM 2–6	*n* of services PPR Travel time	Y	Y	Y	Y	NA	NA	Y	Y
McGrail [77]	2014	Primary health	GP services	National: (SA1 rural; (SA2 metro)	MM 1–7	Travel time	Y	Y	Y	Y	NA	NA	Y	Y
McGrail [16]	2015	Primary health	GP services	National: (SA1 rural; (SA2 metro)	MM 1–7	*n* of PPR Travel time	Y	Y	Y	Y	NA	NA	Y	Y
McGrail [78]	2012	Primary health	GP services	State: CD	MM 2–6	*n* of services PPR Travel time	Y	Y	Y	Y	NA	NA	Y	Y
McGrail [79]	2009	Primary health	GP services	State: CD	MM 2–6	Travel time	Y	Y	Y	Y	NA	NA	Y	Y
McGuire [80]	2011	Primary health	Dental services	City: CD	MM 1	Distance	Y	Y	Y	Y	Y	U	Y	Y
McIsaac [81]	2015	Primary health	GP services	National: POAs	MM 1–7	PPR	Y	Y	Y	Y	Y	Y	Y	Y
Njue [82]	2021	Primary health	Community health	State: SA2	MM 1–7	Distance	Y	Y	Y	Y	Y	N	Y	Y
O'Keefe [83]	2018	Specialist care	Alcohol and other drug treatment services	City: PC	MM 1	Distance	Y	Y	Y	Y	Y	Y	Y	Y
O'Sullivan [84]	2016	Primary health	GP services	National: Regional towns	MM 1–7	Distance *n* of services	Y	Y	Y	Y	U	U	Y	Y
Panaretto [85]	2017	Primary health	Aboriginal and Torres Strait Islander health services	State: LGA, SA2, SLA	MM 1–7	Travel time	Y	Y	Y	Y	N	N	Y	Y
Patel [86]	2019	Primary health	Dental services	City: SA1	MM 1	*n* of practices Distance	Y	U	Y	Y	Y	N	Y	Y
Perera [87]	2010	Primary health	Dental services	State: CD	MM 1–7	*n* of practices Distance	Y	Y	Y	Y	Y	N	Y	Y
Rocha [88]	2013	Primary health	Dental services	City: CD	MM 1	Distance	Y	U	Y	Y	Y	N	Y	Y
Rocha [89]	2013	Primary health	Dental services	City: CD	MM 1	Distance	Y	U	Y	Y	Y	N	Y	Y
Rocha [90]	2015	Primary health	Dental services	City: CD	MM 1	*n* of bus stops to service	Y	U	Y	Y	Y	N	Y	Y
Roeger [91]	2010	Primary health	GP services	City: MB	MM 1	PPR Distance	Y	Y	Y	Y	Y	Y	Y	Y
Rolfe [92]	2017	Hospitals	Maternity services	National: SLA, CD	MM 2–7	Travel time *n* of services	Y	Y	Y	Y	Y	Y	Y	Y
Scott [93]	2006	Primary health	GP services	State: CD	MM 2–7	PPR Distance	Y	Y	Y	Y	Y	N	Y	Y
Sharma [94]	2016	Specialist care	Cancer treatment	State: MB	MM 2–7	Travel time Distance	Y	Y	Y	Y	Y	U	Y	Y
Sharwood [95]	2021	Hospitals	Specialist inpatient service	State: SA3	MM 1–7	Travel time Distance	Y	Y	Y	Y	Y	Y	Y	Y
Shiika [96]	2015	Primary health	Dental services	National: CD excl capital cities)	MM 1–7	Distance *n* of practices	Y	U	Y	Y	Y	N	Y	Y
Shukla [24]	2015	Specialist care	Cancer treatment	State: LGA	MM 1–7	Distance	Y	Y	Y	Y	Y	Y	Y	Y
Siopis [97]	2020	Primary health	Allied Health	National: PC	MM 1–7	PPR	Y	Y	Y	Y	Y	N	Y	Y
Song [98]	2018	Hospitals	Public hospitals	National: LGA	MM 1–7	Travel time	Y	Y	Y	Y	Y	Y	Y	U
Sutarsa [99]	2021	Specialist care	Mental health services	National: LGA	MM 1–7	PPR	Y	Y	Y	Y	Y	N	Y	U
Taylor [100]	2021	Primary health	GP services	Non-metro State: SA2	MM 2–7	Distance	Y	Y	Y	Y	N	N	Y	Y
Tennant [101]	2013	Primary health	Dental services	State: CD, Suburb	MM 1–7	PPR	Y	Y	Y	Y	N	N	Y	Y
Toms [102]	2020	Primary health	GP services	Region: SA1	MM 1–4	PPRDistance	Y	Y	Y	Y	Y	Y	Y	Y
van Gaans [103]	2016	Specialist care	Cardiac services	National: CD	MM 1–7	Distance Travel time	Y	Y	Y	Y	N	N	Y	Y
Verdon [104]	2014	Primary health	Allied Health	National: PC	MM 1–7	*n* of services	Y	Y	Y	Y	N	N	Y	Y
Weerasinghe [105]	2010	Hospitals	Specialist inpatient service	State: LGA	MM 1–7	Distance	Y	Y	Y	Y	Y	Y	Y	Y
Willie-Stephens [106]	2014	Primary health	Dental services	State: CD	MM 1–7	Distance*n* of practices	Y	U	Y	Y	Y	N	Y	Y
Zainab [107]	2015	Primary health	Dental services	City: CD	MM 1	Distance	Y	U	Y	Y	Y	N	Y	Y
Zainol [108]	2016	Primary health	Community health	National: SA2	MM 1–7	*n* of services	Y	Y	Y	Y	N	N	Y	U

Key: *PPR* provider-to-population ratio, *n* number, *MMM* Modified Monash Model, *GP* General practice, *SLA* Statistical Local Areas, *CD* Census Collectors District, *SA3* Statistical Area 3, *SA2* Statistical Area 2, *SA1* Statistical Area 1, *MB* Mesh Blocks, *POA* Postal Areas, *PC* Postcode, *LGA* Local Government Areas, *SSC* State Suburb, *PHN* Primary Health Network

^**a**^ Quality appraisal: 1) Inclusion criteria; 2) Settings; 3) Exposure; 4) Condition; 5) Confounding factors; 6) Strategies for confounders; 7) Outcome measures; 8) Statistical analysis

The geographic scope of the 82 articles included national (*n* = 33; 40%), state (*n* = 27; 33%), metropolitan areas (*n* = 18; 22%), and specified regional/rural/remote areas (*n* = 4; 5%) (Table 4). Most articles used distance-based measures, including travel time (*n* = 30; 37%) and travel distance along a road network (*n* = 21; 26%), and Euclidean distance (*n* = 24; 29%). Articles that included area-based measures include provider-to-population ratio (*n* = 16; 20%), the number of services (*n* = 10; 12%) or practices (*n* = 8; 10%) per defined geographic area.

**Table 4:** Geographical scale of articles analysing health service accessibility

Geographical scale	Health service discipline	
National	Primary health:	• Dental services (*n* = 10) [41, 48, 52, 57–61, 71, 96] • GP services (*n* = 4) [16, 77, 81, 84] • Allied health (*n* = 3) [74, 97, 104] • Community health (*n* = 1) [108] • Other primary care services (*n* = 1) [32]
	Specialist care:	• Cardiac services (*n* = 4) [34, 35, 37, 103] • Mental health services (*n* = 1) [99] • Renal disease management (*n* = 1) [49] • Palliative care services (*n* = 1) [40]
	Hospitals:	• Intensive care services (*n* = 2) [44, 45] • Maternity services (*n* = 2) [53, 92] • Public hospitals (*n* = 1) [98] • Acute cardiac care (*n* = 2) [36, 38]
State	Primary health:	• GP services (*n* = 5) [75, 76, 78, 79, 93] • Dental services (*n* = 4) [65, 87, 101, 106] • Community health (*n* = 1) [82] • Pharmacy (*n* = 1) [70] • Allied health (*n* = 1) [69] • Aboriginal and Torres Strait Islander health services (*n* = 2) [68, 85]
	Hospitals:	• Specialist inpatient service (*n* = 2) [95, 105] • Outpatient clinic (*n* = 2) [42, 95] • Emergency care (*n* = 2) [31, 51]
	Specialist care:	• Cancer treatment (*n* = 6) [24, 39, 46, 54, 55, 94]
	Health promotion and prevention:	• Infectious disease screening (*n* = 1) [66]
Metropolitan area	Primary health:	• Dental services (*n* = 8) [29, 30, 80, 86, 88–90, 107] • GP services (*n* = 4) [33, 56, 73, 91] • Allied health (*n* = 1) [47] • Other primary care services (*n* = 1) [72]
	Specialist care:	• Alcohol and other drug treatment services (*n* = 1) [83] • Palliative care (*n* = 1) [67]
	Health promotion and prevention:	• Cancer screening (*n* = 2) [62, 63]
Regional area or centre	Primary health:	• GP services (*n* = 3) [43, 84, 100]
	Hospitals:	• Emergency care (*n* = 1) [64]

The geographic classifications applied varied across the articles (Table 5), including spatial classifications from the Australian Bureau of Statistics (ABS) Main Structure (*n* = 56) and Non-ABS Structure (*n* = 23). The ABS Main Structure is used to analyse a broad range of social, demographic, and economic statistics for states, territories, and statistical areas. In contrast, the Non-ABS Structures are administrative regions not defined or maintained by the ABS, such as local government areas and postal areas [109]. Fourteen articles (17%) included data analyses from the rural context (e.g., stratified by remoteness), with only four focusing specifically on a regional area or centre; the remaining articles were at a national or state level, disaggregating rural populations according to remoteness categories. To classify remoteness, articles used the ABS classifications, such as Australian Standard Geographic Classification-Remoteness Area (ASGC-RA) (*n* = 4) and the more recent, Australian Statistical Geography Standard–Remoteness Area (ASGS-RA) (*n* = 15). Remoteness was also classified using ARIA (*n* = 2) and ARIA + (*n* = 12). Nine articles used a General Post Office (GPO) as the central datum point and applied a range of buffers to classify metropolitan areas, including 25 km (*n* = 1), 50 km (*n* = 7), and 100 km (*n* = 1). None of the included articles used the Modified Monash Model (MMM) to classify geographic areas. The following section provides an overview of articles from each health service area.

**Table 5:** Overview of geographic classifications used to analyse accessibility across health service areas

**Geographic classifications**		**Health service area**
**Australian Bureau of Statistics (ABS) Main Structure (*n* = 56)**		
* Australian Standard Geographical Classification (ASGC)*		
SLA	Specialist care:	• Cancer treatment (*n* = 3) [39, 54, 55]
	Primary health:	• Aboriginal and Torres Strait Islander health services (*n* = 1) [85]
CD	Primary health:	• Dental services (*n* = 12) [29, 30, 65, 80, 87–90, 96, 101, 106, 107] • GP services (*n* = 7) [56, 75, 76, 78, 79, 93]
	Specialist care:	• Cardiac services (*n* = 4) [34, 35, 37, 103]
	Hospitals:	• Maternity services (*n* = 1) [92] • Acute cardiac care (*n* = 2) [36, 38]
* Australian Statistical Geography Standard (ASGS)*		
SA3	Hospitals:	• Specialist inpatient services (*n* = 1) [95]
	Primary health:	• Dental services (*n* = 1) [48]
	Specialist care:	• Renal disease management (*n* = 1) [49]
SA2	Primary health:	• Dental services (*n* = 5) [41, 52, 57, 58, 60] • Community health (*n* = 2) [82, 108] • GP services (*n* = 1) [100] • Allied health (*n* = 1) [69] • Aboriginal and Torres Strait Islander health services (*n* = 1) [68]
SA1	Primary health:	• Dental services (*n* = 4) [59, 61, 71, 86] • GP services (*n* = 4) [16, 43, 77, 102] • Allied health (*n* = 1) [47]
	Specialist care:	• Palliative care (*n* = 1) [67]
	Hospitals:	• Emergency care (*n* = 1) [51]
MB	Specialist care:	• Cancer treatment (*n* = 2) [46, 94]
	Health promotion and prevention:	• Infectious disease screening (*n* = 1) [66]
	Primary health:	• GP services (*n* = 1) [91]
**Non-ABS structures (*n* = 23)**		
POAs	Primary health:	• GP services (*n* = 2) [73, 81]
PC	Primary health:	• Allied health (*n* = 3) [74, 97, 104] • GP services (*n* = 1) [84] • Pharmacy (*n* = 1) [70] • Other primary care services (*n* = 1) [32]
	Hospitals:	• Intensive care services (*n* = 2) [44, 45] • Outpatient clinic (*n* = 2) [42, 50]
	Specialist care:	• Alcohol and other drug treatment services (*n* = 1) [83] • Palliative care services (*n* = 1) [40]
LGA	Primary health:	• Aboriginal and Torres Strait Islander health services (*n* = 1) [85] • Other primary care services (*n* = 1) [72]
	Specialist care:	• Cancer treatment (*n* = 1) [24] • Mental health services (*n* = 1) [99]
	Hospitals:	• Public hospitals (*n* = 1) [98] • Specialist inpatient services (*n* = 1) [105]
SSC	Health promotion and prevention:	• Cancer screening (*n* = 2) [62, 63]
PHN	Hospitals:	• Emergency care (*n* = 1) [64]

Key: *SLA* Statistical Local Areas, *CD* Census Collectors District, *SA3* Statistical Area 3, *SA2* Statistical Area 2, *SA1* Statistical Area 1, *MB* Mesh Blocks, *POA* Postal Areas, *PC* Postcode, *LGA* Local Government Areas, *SSC* State Suburb, *PHN* Primary Health Network

### Primary health services

#### Dental services

Twenty-two articles, published between 2010 to 2022, focused on accessibility to public and private dental practices, dental hospitals, and distribution of the dental workforce [29, 30, 41, 48, 52, 57–61, 65, 71, 80, 86–90, 96, 101, 106, 107]. Articles focused on specific target populations, including paediatric [30, 59], > 65 years [61, 86], economically disadvantaged [41], and rural, regional, or remote [48, 60, 87, 96]. One article analysed dental services for people living with a disability and used the National Survey of Disability, Ageing and Carers to model the prevalence of disability [71]. Sixteen articles accounted for socio-economic status (SES), seven used the ABS Socio-Economic Indexes for Areas (SEIFA) [30, 80, 88–90, 96, 106], and nine articles further specified the index used; for example, the Index of Relative Socio-economic Disadvantage (IRSD) [29, 52, 57–59, 61, 65, 86, 87]. Five articles used standardised geographic classification systems to define remoteness areas, including ARIA + [57–60] and the ABS ASGS-RA [48]. Nine articles used proximity around a GPO to define a metropolitan area with varying buffer distances, including 25 km [29], 50 km [86, 88–90, 96, 106, 107], and 100 km [71]. Physical access to dental services was analysed using both area- and distance-based measures. Area-based measures include the number of practices per defined area [52, 61, 65, 71, 86, 87, 96, 106] and PPR [57, 58, 60, 101]. Distance-based measures include Euclidean distance or multiple ring buffer [29, 30, 41, 65, 71, 80, 86–90, 96, 106, 107], travel time using road networks [48, 59], and public transport networks [86, 90]. Definition of spatial methods are summarised in the supplementary materials (see Supplementary File 3).

#### General practice services

Sixteen articles, published between 2003 to 2021, focused on accessibility to general practice (GP) services [16, 33, 43, 56, 73, 75–79, 81, 84, 91, 93, 100, 102]. Of those articles, three were from the same study [75, 76, 79]. Articles focused on specific target populations, including antenatal [33], > 65 years [100], patients with chronic disease [73], and rural, regional, or remote [75, 76, 78, 79, 84, 93, 100]. Ten articles accounted for SES status using the ABS SEIFA [56, 75, 76, 79], with six articles further specifying the index used, including the Index of Relative Socio-economic Advantage and Disadvantage (IRSAD) [91] and IRSD [16, 33, 43, 73, 102]. Seven articles used standardised geographic classification systems to define remoteness areas, including ARIA [81], ABS ASGC-RA [91], and ABS ASGS-RA [16, 43, 73, 77, 100]. Physical access to GP services was analysed using both area- and distance-based measures. Area-based methods that were used include the two-step floating catchment area (2SFCA) method [16, 75–79, 91, 102] and PPR [73, 81, 93]. Distance-based measures include travel distance calculated using road networks [33, 43, 56, 84, 100] and travel time using road networks [16, 75–79]. Articles included methods of spatial analyses, such as regression, simulations, cluster detection, network analysis, and spatial autocorrelation.

#### Community health services

Two articles, published in 2016 and 2021, focused on accessibility to community health services, including healthcare facilities [108], and child and family health services [82]. Articles focused on specific target populations, including > 65 years [108] and migrant and refugee populations [82]. One article used the ABS standardised geographic classification system to define remoteness areas (ASGS-RA) [108]. Physical access to community health services was analysed using both area- and distance-based measures. Area-based and distance-based measures were the number of services per defined geographic area [108] and travel time using the road network [82], respectively. One article applied the Local Indicator Spatial Association (LISA) technique to analyse the distribution of the population aged over 65 years in relation to community healthcare facilities [108].

#### Allied health services

Five articles, published between 2014 to 2020, focused on accessibility to allied health services, including speech pathology [74, 104], disability and rehabilitation [47, 69], and dietetics [97]. Articles focused on specific target populations, including paediatric [74, 104], patients with diabetes [97], and patients with a disability [47, 69]. One article used ARIA + to define remoteness areas [69], three articles were nationally focused and did not classify remoteness areas [74, 97, 104], and one article was metropolitan focused [47]. Physical access to allied health services was analysed using both area- and distance-based measures. Area-based measures include the number of services [47, 74, 104] or providers [97] per defined geographic area. Distance-based measures include road network distance [47] and travel time using road network [69]. Spatial analyses varied across the articles; two articles created an origin–destination cost matrix [47, 69], and another analysed spatial clusters using hot spot analyses and cluster and outlier detection [69].

#### Pharmacy services

One article, published in 2021, analysed access to opioid substitution treatment (OST) pharmacies across South Australia for public and private OST patients [70]. Physical access to pharmacy services from patients’ locations was analysed using distance-based measures, such as Euclidean distance and travel time using road networks. The article produced density maps for hot spot analyses.

#### Aboriginal and Torres Strait Islander health services

Two articles, published in 2017 and 2021, focused on accessibility to Aboriginal and Torres Strait Islander health services, including community-controlled primary healthcare services [68, 85]. Both articles used the ABS standardised geographic classification system to define remoteness areas (ASGS-RA) and distance-based measures such as travel time using the road network. One article created an origin–destination cost matrix framework to estimate travel times and undertook hotspot analysis [68].

#### Other primary care services

One article, published in 2018, focused on accessibility to diabetic health services, including GP, dieticians, endocrinologists, and diabetic educators [72]. The article utilised 10,000 random residential address points across metropolitan Melbourne and created two origin–destination cost matrix frameworks to estimate travel times between the synthetic address point to the nearest health service for private and public transportation. Another article, published in 2010, examined accessibility to HIV medical services [32]. The article used ARIA to classify remoteness areas and travel distance.

### Specialist health services

#### Cancer treatment services

Six articles, published between 2012 to 2016, focused on accessibility to cancer treatment services [39, 46, 54, 55, 94, 110]. Articles focused on specific target populations, including patients with specific types of cancer; for example, breast [39, 54, 55, 94], colorectal [39], and prostate cancer [94]. Three articles accounted for SES using the ABS IRSAD [54, 55] and IRSD [39]. One article used ARIA + to define remoteness areas [55], another used the cancer-specific remoteness index, TRAvel to Cancer Treatment (TRACT) [54], and the remaining articles did not define remoteness areas. Physical access to cancer treatment services was analysed using distance-based measures, including Euclidean distance or multiple ring buffer [46, 55, 94, 110], road network distance [110], and travel time using road network [39, 54, 94]. Articles undertook a range of spatial analyses, consisting of regression [46, 54, 110], Bayesian spatial survival models [39, 54, 55], simulation models [39], and global clustering [39].

#### Cardiac services

Four articles, published between 2007 to 2016, focused on accessibility to cardiac services [34, 35, 37, 103]. Articles focused on specific target populations, including > 45 years [37] and patients with chronic heart failure accessing cardiac rehabilitation or management services [34, 35, 103]. Two articles used the ARIA/ARIA + to define remoteness areas [34, 35]. Physical access to cardiac services was analysed using both area-based and distance-based measures. Two articles used the number of services within a defined geographic area as an area-based measure [34, 37]. Most articles used distance-based measures, including road network distance [35, 37, 103] and travel time along a road networks [37, 103]. One article applied network analysis and raster based-cost distance modelling [103].

#### Other specialist services

One article, published in 2020, analysed the spatial distribution of mental health nurses across Australia [99]. Physical access to mental health services was analysed using the area-based measure, PPR, by examining the total number of mental health nurses per 100,000 persons and used ARIA + to define remoteness areas. Another article published in 2018, analysed access to needle and syringe dispensing outlets across Melbourne, Victoria, for participants recruited into the Melbourne injecting drug user cohort (MIX) study [83]. Physical access to needle and syringe dispensing outlets was analysed using Euclidean distance. Two articles, published in 2012 and 2020, analysed access to palliative care services [40, 67]. Physical access to palliative care services was analysed using distance-based measures, including Euclidean distance [40] and travel time [67]. One article published in 2020, analysed access to renal disease management services across Australia [49]. Physical access to renal services was analysed using the distance-based measure, travel time, and used the ABS ASGS-RA to classify remoteness areas.

### Hospital services

Fourteen articles, published between 2010 to 2021, focused on accessibility to public hospitals [98] and hospital services, including specialist inpatient care [95, 105], outpatient clinics [42, 50], maternity care [53, 92], intensive care [44, 45], emergency care [31, 51, 64], and acute cardiac care [36, 38]. Of those articles, two were from the same study investigating intensive care [44, 45], and two were from the same study relating to the development of the Cardiac Access-Remoteness Index of Australia (Cardiac ARIA) [36, 38]. Four articles accounted for socio-economic status using the ABS SEIFA [95, 105], with two articles further specifying the index, IRSAD [50] and IRSD [92]. Nine articles used standardised geographic classification systems to define remoteness, including ARIA + [36, 38, 44, 45], ABS ASGC-RA [64, 92], and ABS ASGS-RA [42, 53, 98]. Physical access to hospital services was analysed using both area- and distance-based measures. Area-based measures were primarily used for analysing maternity services and included the number of services per defined geographic area [53, 92]. Distance-based measures were used in the remaining articles and included Euclidean distance or multiple ring buffers [44, 45, 105], road network distance [36, 38, 42, 64], and travel time using a road network [31, 36, 38, 50, 51, 95, 98]. One article applied a modified kernel density two-step floating catchment area (MKD2SFCA) model to compute accessibility of travel times, in addition to examining the spatial and temporal variations of the hot spot analyses with LISA [98]. Two articles undertook spatial analyses, including network analysis and raster-based-cost distance modelling [36, 38].

### Health promotion and prevention

#### Infectious disease testing sites

One article, published in 2021, analysed the accessibility of SARS-CoV-2 point-of-care-test (POCT) site locations across Victoria, using the ASGS-RA to define remoteness areas across the state [66]. The article accounted for SES using the ABS SEIFA IRSD. The article used distance-based measures, such as travel time along road networks. Inferential analysis was undertaken to analyse travel times to the closest POCT site across remoteness areas.

#### Cancer screening services

Two articles from the same study, published in 2021, focused on breast cancer screening (BCS) venue location features and utilisation across Greater Sydney, NSW [62, 63]. Distance was measured along the road network and used to analyse physical access from patients’ residential postcodes to the BCS venue locations. Both articles conducted hot spot analyses to assess spatial clustering. The articles examined residential-area socio-demographic characteristics using multiple measures, including age, language, education, employment, and motor vehicle ownership, instead of a composite index, such as the ABS SEIFA.

#### Quality appraisal

Twenty-one articles (27%) met all 8 criteria, 55 articles (68%) met 6–7 criteria, and 6 articles (5%) met 5 criteria (Table 4). Inclusion criteria, conditions, and outcome measures were met in all articles. Settings described were met for 68 articles (83%), with 14 articles unclear (17%), mostly due to methods of classifying remoteness areas. Most articles (99%) used valid or reliable measures for exposure. Fifty-two articles (63%) identified confounding factors, and 22 (27%) explicitly addressed confounding factors with statistical adjustments, stratifications, and model selection. Seventy-seven articles (83%) used appropriate statistical analyses to address their research aims. Finally, the reliability or validity of the GIS methods was unclear in 5 articles (5%), primarily due to unclear geocoding methods or information about the software used.

## Discussion

This is the first review to synthesise the Australian peer-reviewed literature identifying how physical access to health services has been measured and accounted for over the past 20 years. Findings demonstrated that although a relatively large number of articles have analysed access, the research mostly focused at national and state levels. Only 14 out of 82 (17%) articles specifically assessed access in rural areas, despite 28.7% of the Australian population at the 2016 census, living in rural areas and being the most disadvantaged in terms of geographic access to health services [111, 112].

There was substantial heterogeneity in the objective physical access measures and geographic classifications used to examine spatial access to health services, particularly across health disciplines. The majority of articles focused on measuring access to primary care services, such as dentistry and GP services, as opposed to mental health, nursing, and allied health services, despite reports of the disproportionate distribution of these services across Australia [111, 113–115]. As allied health encompasses diverse disciplines, the increased complexity of allied health data makes comprehensive workforce analyses more difficult, in the absence of national surveys and an allied health central registry. For example, occupational therapists and physiotherapists are regulated by the Australian Health Practitioner Regulation Agency (AHPRA) [116]. However, dietitians are regulated by Dietitians Australia as a member of the National Alliance for Self Regulating Health Professionals [117], which means allied health service provision data are not centrally located or easily accessible. Allied health workforce, services and location data collection will remain fragmented and will unlikely be representative of the actual workforce unless investment is made in more nationally streamlined regulation and data collection systems [118]. The Nursing and Allied Health Graduate Outcome Tracking (NAHGOT) study is a research collaboration that will address this gap in health workforce data by tracking graduates, providing a more comprehensive understanding of workforce trends over time that can inform planning [119, 120].

Technological advancements in analysis software and changing indices of remoteness over time have increased the capability to spatially examine health service access; however, this review identified that consistent access measures are not established in the Australian context. The use of distance-based measures appears to be shifting towards more sophisticated measures. For example, articles published before 2015 primarily used Euclidean distance; however, articles published since then have used distance calculations and travel times along road networks. Areas of Australia face challenges when reliably applying distance-based measures, such as those areas affected by the monsoon season. For instance, precipitation and its duration can affect vehicle speed and may result in road closures, impacting the travel time-determined spatial accessibility [121]. Only one included article considered weather conditions when accessing health services and used remote sensing data for monthly precipitation rates when estimating variations in traffic speed. [98] Spatial access to healthcare services are a strong predictor of health disparities [13]. For example, previous reviews have examined the relationship between transport accessibility and health outcomes, observing an association between travelling further and having poorer health outcomes, highlighting the importance of distance when considering health service access [122].

Practice-to-population and PPR were the most common area-based measures predominantly used to analyse primary health accessibility. This might be explained by the use of similar measures nationally, as the AIHW reports on the primary health workforce in Australia by the number of full-time equivalent (FTE) health professionals per 100,000 population (FTE rate) [123]. Dental services primarily used practice-to-population distribution; however, this method does not account for the number of providers within a practice, average hours worked, appointment availability or wait times [58, 87]. Severe limitations exist when using PPR to measure the distribution of fractional services, such as outreach, locum, or mobile services, often utilised in rural and remote areas [124]. For instance, service patterns and the supply of specialists will vary by regional context [84], making it challenging to measure supply ratios reliably, with implications for workforce policy and planning.

Accessibility and distributional fairness of health services is an important goal for health planners and policy makers. However, any type of distributional inequality of services is not necessarily considered spatial inequity, as it is dependent on the health needs of a population [125, 126]. Equitable distribution requires higher levels of resourcing allocated to high-needs populations (vertical equity) [127]. For instance, population groups with lower SES are at greater risk of poor health and, on average, have higher primary care needs [128]. This is also the case for rural and remote communities in Australia. A needs-based funding mechanism is a suggested policy solution to ensure equitable resource allocation in these sparsely populated areas [129]. Without data at the individual-level scale, individual health needs and inequalities are difficult to reliably predict or measure, potentially resulting in a mismatch between the supply of health services and the health needs of a community.

Among the dental service access research, practice distribution was examined using socio-economic indicators to determine the distribution relative to the socio-economic profile of areas, likely reflective of this sector, as private clinics have been shown to be influenced by the market-driven economy, and most likely survive in wealthier suburbs and more densely populated areas [52, 101]. No other discipline examined practice-to-population distribution using socio-economic indicators, which may be due to the dental profession being funded more by patients and private health insurance when compared to other disciplines. Other privatised services, such as optometry, physiotherapy, or psychology, may show similar results; however, this review did not find articles from these disciplines, and we should not assume this to be the case owing to a range of regulatory and other factors that can influence the choice of location [130]. Research has started to analyse the distribution of allied health workforce across socio-economic areas within Australia [115].

In addition to heterogeneity in physical access measures, this review observed variation in the geographic classification measures used, including the ABS main structure and non-ABS structure. Postcodes and LGAs, were the most common non-ABS spatial units used, and several articles applied the ABS SEIFA. However, the use of SEIFA with large geographic areas, such as LGAs has been criticised [131, 132]. This invokes both the ecological fallacy and MAUP when SEIFA scores for larger geographic areas are used. Due to inherent population heterogeneity and complexity, individual-level characteristics vary from the average area-level characteristics [131, 132]. The application of large artificial boundaries can result in the misidentification of these characteristics [131, 132]. Consistent with other research [23], this review also shows variations in rurality definitions. The lack of a standardised approach further hinders the ability to compare articles and health service access across remoteness areas. This review did not find any articles using the MMM to examine the geographic distribution of health services, despite its national policy relevance [112, 133]. The lack of uptake is likely due to the recent transition (2022) by the Australian Government's Department of Health in adopting the MMM geographical classification for all workforce programs, research and translation, and service delivery [112].

Analysing access at the finest resolution is often limited by the availability and quality of the data acquired [134], such as withheld data in private sectors and data restricted by privacy laws, such as access to population data for small areas. Nordic countries have a long tradition of systematically collecting individual-level population data through mandated government-maintained nationwide public registries to generate accurate data, guide decision-making, and improve the health and welfare of the population [135, 136]. High levels of trust in public institutions enable the linkage of this national data [137]. Australia has a complex health system, with some aspects controlled and funded by the Commonwealth Government and others managed by each State and Territory Government, resulting in population data being held by separate agencies [138]. In addition to the health system, Australia has a complex authorising environment, with each jurisdiction implementing legislation and related policies and practices [138]. In the absence of individual-level data, using a synthetic population at the small area level may overcome data gaps while maintaining privacy and confidentiality laws.

Measuring access contributes to a broader understanding of the performance of health systems within and between countries; for instance, the Commonwealth Fund compares health system performance among Australia and other high-income countries based on healthcare access [2]. The Commonwealth Fund defines *access* to care as affordability and timeliness, whereby Australia is a high-performing country. However, inequities exist regarding the spatial accessibility of primary health care services matching the health requirements of communities, especially in rural and remote Australia [129]. The health needs are known to be heterogeneous across remoteness. They are difficult to measure—due to differences in sample size, demographics, and investment in these communities—making health needs challenging to decipher. For example, very remote areas can be under-represented in population health surveys, thus, limiting the availability of information about the health needs of these communities [139]. There is a need for a national policy designed to optimise access to health services according to health service requirements to maximise equity. Combining data regarding a population's health needs and using a standardised approach to measuring travel times, such as road networks and modelled distance/time calculations, could better inform such health policy and guide workforce planning.

### Implications for practice

There is a need for more standardised accessible data for different health disciplines, such as allied health, which play a key role in healthcare delivery to support further research. Regulators and governments need to consider appropriate and accessible data collection on these services. While a shift to the MMM definition of rurality may generate national comparisons, it is still subject to the MAUP and could potentially mask health service access issues. This is especially the case in more remote locations where the spatial units are considerably larger to encompass the required population to maintain consistency across Australia. Several steps that need to be taken to ensure the issue of access to health services can be properly measured and understood, with the first step defining access. The second step is moving to an address-based spatial unit and, where possible, using road networks and modelled distance/time calculations. The third step is appropriate appraisal tools for assessing the methodological quality of health geography and spatial research.

### Strengths and limitations

To our knowledge, this review is the first comprehensive literature synthesis using a systematic review methodology to examine the evidence on how spatial measures are applied to understand health service accessibility in Australia. A strength of this study is the use of comprehensive and broad search terms in multiple databases from the past 20 years. Given the long-term investment by the Australian Government in national policies such as the Rural Health and Multidisciplinary Training Program (focused on addressing workforce maldistribution and ultimately improving healthcare access) [140], this review is complementary as it expands the evidence base for a better understanding of this challenge. There were several limitations of this review. Firstly, the lack of appropriate quality appraisal tools to assess the methodological quality of spatial research, resulting in reduced reliability of the assessment of systematic errors. Secondly, this review does not go beyond spatial access (geographic accessibility). Aspatial access (e.g., affordability, timeliness, accommodation, acceptability, and awareness) are also important when considering access to health services. However, the rationale for addressing the spatial dimensions first is that without considering availability and accessibility, the service cannot be utilised [10, 11, 112]. Future research may consider focusing on how other dimensions of access are examined in the Australian context. Thirdly, whilst a systematic review methodology is robust, it is limited when translating complex problems, such as rural and remote health issues, into policy [141]. Other types of reviews (e.g., realist, narrative, scoping) might be required to gain a deeper understanding of the issues [142].

## Conclusion

Objective and transparent access measures that are fit for purpose are imperative to address persistent health inequities and inform equitable resource distribution and evidence-based policymaking. This review identified substantial heterogeneity in the spatial measures and geographic classifications used to examine access to health services. Although primary health services were the most studied area, there are gaps where more research is required, such as mental health, nursing, and allied health services. Future research should aim to analyse access at the finest resolution and, where possible and appropriate, aim to use standardised approaches to classifying rural and remote populations relevant to the purpose of the study. Our study supports the need for a consensus on what constitutes reasonable access to different health services, thereby improving the ability to interpret spatial access for policy purposes.

## Supplementary Information

**Additional file 1: Supplementary File 1.** PRISMA Checklist for ‘The application of spatial measures to analyse health service accessibility in Australia: a systematic review and recommendations for future practice.

**Additional file 2: Supplementary File 2.** Search Strategy.

**Additional file 3: Supplementary File 3. **Definition of Spatial Methods*.*

## Data Availability

All supporting data are included in this published article and its Supplementary files. No primary data were collected.

## Abbreviations

ABS: Australian Bureau of Statistics
AIHW: Australian Institute of Health and Welfare
ARIA: Accessibility/ Remoteness Index of Australia
ASGC-RA: Australian Standard Geographical Classification-Remoteness Area
ASGS-RA: Australian Statistical Geography Standard–Remoteness Area
AHPRA: Australian Health Practitioner Regulation Agency
BCS: Breast cancer screening
CD: Census Collectors District
CVD: Cardiovascular disease
FTE: Full-time equivalent
GP: General practice
GPO: General Post Office
IRSAD: Index of Relative Socio-economic Advantage and Disadvantage
IRSD: Index of Relative Socio-economic Disadvantage
LGA: Local Government Areas
LISA: Local Indicator Spatial Association technique
MAUP: Modifiable areal unit problem
MB: Mesh Blocks
MKD2SFCA: Modified kernel density two-step floating catchment area
MMM: Modified Monash Model
NSW: New South Wales
NAHGOT: Nursing and Allied Health Graduate Outcome Tracking
OST: Opioid substitution treatment
PC: Postcode
PHN: Primary Health Network
POA: Postal Areas
POCT: Point-of-care-test
PPR: Provider-to-population ratio
PRISMA: Preferred Reporting Items for Systematic Reviews and Meta-analyses
SA3: Statistical Area 3
SA2: Statistical Area 4
SA2: Statistical Area 2
SA1: Statistical Area 1
SEIFA: Socio-Economic Indexes for Areas
SES: Socio-economic status
SLA: Statistical Local Areas
TRACT: TRAvel to Cancer Treatment
SSC: State Suburb
2SFCA: Two-step floating catchment area
