# Supplementary material for: The application of spatial measures to analyse health service accessibility in Australia: a systematic review and recommendations for future practice
Source: BMC Health Serv Res. 2023 Apr 1;23:330. doi: 10.1186/s12913-023-09342-6 (PMC10066971; doi:10.1186/s12913-023-09342-6)
Supplement: Supplementary file 2 — Additional file 2: Supplementary File 2. Search Strategy. [file 12913_2023_9342_MOESM2_ESM.docx]

**Supplementary File 2. Search Strategy**

| **SEARCH STRATEGY – MEDLINE, CINAHL, EBSCOHOST** |
| --- |
| Australia* OR “New South Wales” OR “Northern Territory” OR Queensland OR Victoria OR Tasmania |
| **AND** |
| Patient OR patients OR hospital OR hospitals OR “emergency department” OR “trauma centre” OR ICU OR radiotherapy OR “radiation therapy” OR nurse OR nurses OR nursing OR “general practi*” OR doctor OR pharmacy OR pharmacist OR dental OR dentistry OR ambulance OR “emergency transport” OR “speech pathology*” OR “speech therap*”OR dietician OR dietetic OR physiotherapy* OR TI ((health N2 (service* OR care OR access* OR community OR allied)) OR AB ((health N2 (service* OR care OR access* OR community OR allied)) OR TI ((service* N2 (healthcare OR maternity OR cancer OR oncology OR medical OR disability OR mental health OR delivery OR ambulance)) OR AB((service* N2 (healthcare OR maternity OR cancer OR oncology OR medical OR disability OR mental health OR delivery OR ambulance)) OR TI ((care N2 (primary OR aged OR intensive OR critical OR palliative OR cancer OR specialist OR ambulatory)) OR AB ((care N2 (primary OR aged OR intensive OR critical OR palliative OR cancer OR specialist OR ambulatory)) |
| **AND** |
| “geographic* information system*” OR spatial OR geocod* OR TI ((travel* N2 (distance OR time OR road OR patient) OR AB ((travel* N2 (distance OR time OR road OR patient) OR “Accessibility/Remoteness Index of Australia (ARIA+)” |
| **Limiters:** Date: 2002-2022; Language: English; Geography: Australia |
| **SEARCH STRATEGY – EMBASE** |
| Australia* OR “New South Wales” OR “Northern Territory” OR Queensland OR Victoria OR Tasmania |
| **AND** |
| Patient OR patients OR hospital OR hospitals OR “emergency department” OR “trauma centre” OR ICU OR radiotherapy OR “radiation therapy” OR nurse OR nurses OR nursing OR “general practi*” OR doctor OR pharmacy OR pharmacist OR dental OR dentistry OR ambulance OR “emergency transport” OR “speech pathology*” OR “speech therap*”OR dietician OR dietetic OR physiotherapy* OR (health NEXT/2 (service* OR care OR access* OR community OR allied)):ti,ab OR (service* NEAR/2 (healthcare OR maternity OR cancer OR oncology OR medical OR disability OR mental health OR delivery OR ambulance)):ti,ab OR (care NEAR/2 (primary OR aged OR intensive OR critical OR palliative OR cancer OR specialist OR ambulatory)):ti,ab |
| **AND** |
| “geographic* information system*” OR spatial OR geocod* OR “Accessibility/Remoteness Index of Australia (ARIA+)” OR (travel* NEAR/2 (distance OR time OR road OR patient)):ti,ab |
| **Limiters:** AND [embase]/lim AND [2002-2022]/py AND Australia:ca |
| **SEARCH STRATEGY - SCOPUS** |
| Australia* OR “New South Wales” OR “Northern Territory” OR Queensland OR Victoria OR Tasmania |
| **AND** |
| Patient OR patients OR hospital OR hospitals OR “emergency department” OR “trauma centre” OR ICU OR radiotherapy OR “radiation therapy” OR nurse OR nurses OR nursing OR “general practi*” OR doctor OR pharmacy OR pharmacist OR dental OR dentistry OR ambulance OR “emergency transport” OR “speech pathology*” OR “speech therap*”OR dietician OR dietetic OR physiotherapy* OR “health service” OR “healthcare” OR “health care” OR “community health” OR “allied health” OR “maternity service*” OR “cancer service*” OR “oncology service*” OR “medical service*” OR “disability service*” OR “ambulance service*” OR “primary care” OR “aged care” OR “intensive care” OR “emergency medicine” OR “critical care” OR “palliative care” OR “specialist care” |
| **AND** |
| “geographic* information system*” OR spatial OR geocod* OR “Accessibility/Remoteness Index of Australia (ARIA+)” |
| **Limiters:** Date: 2002-2022; Language: English; Geography: Australia |
| **SEARCH STRATEGY – WEB OF SCIENCE** |
| Australia* OR “New South Wales” OR “Northern Territory” OR Queensland OR Victoria OR Tasmania |
| **AND** |
| Patient OR patients OR hospital OR hospitals OR “emergency department” OR “trauma centre” OR ICU OR radiotherapy OR “radiation therapy” OR nurse OR nurses OR nursing OR “general practi*” OR doctor OR pharmacy OR pharmacist OR dental OR dentistry OR ambulance OR “emergency transport” OR “speech pathology*” OR “speech therap*”OR dietician OR dietetic OR physiotherapy* OR health NEXT/2 (service* OR care OR access* OR community OR allied) OR service* NEAR/2 (healthcare OR maternity OR cancer OR oncology OR medical OR disability OR delivery OR ambulance) OR care NEAR/2 (primary OR aged OR intensive OR critical OR palliative OR cancer OR specialist OR ambulatory) |
| **AND** |
| “geographic* information system*” OR spatial OR geocod* OR travel* NEAR/2 (distance OR time OR road OR patient) OR “Accessibility/Remoteness Index of Australia (ARIA+)” |
| **Limiters:** AND [embase]/lim AND [2002-2022]/py AND Australia:ca |
