# Supplementary material for: The application of spatial measures to analyse health service accessibility in Australia: a systematic review and recommendations for future practice
Source: BMC Health Serv Res. 2023 Apr 1;23:330. doi: 10.1186/s12913-023-09342-6 (PMC10066971; doi:10.1186/s12913-023-09342-6)
Supplement: Supplementary file 3 — Additional file 3: Supplementary File 3. Definition of Spatial Methods. [file 12913_2023_9342_MOESM3_ESM.docx]

**Supplementary File 3. Definition of Spatial Methods**

| **Approach** | **Definition** |
| --- | --- |
| **Area-based measures** | |
| *n* of practices or services | Number of practices or health services within a defined geographic area. |
| PPR | Supply ratio of the population to healthcare providers within a defined geographic area [21]. |
| Floating catchment area models (e.g., 2SFCA, 3SFCA) | Multi-step model that uses population service catchment areas to calculate a supply-to-demand ratio for each health service location [59]. |
| **Distance-based measures** | |
| Euclidean distance | Straight-line distance between two points [15]. |
| Multiple ring buffer | Multiple buffers at specified distances around the health service. |
| Network distance | The distance (m, km) between a point of origin and the nearest health service travelling along a road or public transport network [13]. |
| Travel time | The time travelled (min, hr) along a road network between a point of origin and health service [13]. |
| **Spatial analysis** | |
| Origin Destination Cost Matrix | Measures the least-cost (travel time and travel distance) paths along the network from multiple origins to multiple destinations [111]. |
| Raster based-cost distance modelling | Distance to the nearest source for each cell in the raster, based on the least-accumulative cost over a cost surface [112]. |
| Spatial autocorrelation | Calculates the correlation within variables across a global or local georeferenced space [113]. Global spatial autocorrelation analyses the characteristics across the entire study area (e.g., clustering) and summarises the overall pattern of spatial dependence in the data into a single indicator, such as Moran’s I and Geary’s C statistics [114].  Local Indicators of Spatial Association (LISA) statistics identify individual-level characteristics and possible centres of statistically significant clustering (e.g., hot spots or cold spots) [115]. |
| Hotspot analysis | Identifies statistically significant spatial clusters of high values (hot spots) and low values (cold spots). |
| Cluster and outlier detection | Identifies concentrations of high values, concentrations of low values, and spatial outliers. |

Key: *n* = number; PPR = provider-to-population ratio; 2SFCA = Two-step floating catchment area; 3SFCA = Three-step floating catchment area; m = metre; km = kilometre; min = minute; hr = hour
